# Large scale real-time PCR validation on gene expression measurements from two commercial long-oligonucleotide microarrays

**DOI:** 10.1186/1471-2164-7-59

**Published:** 2006-03-21

**Authors:** Yulei Wang, Catalin Barbacioru, Fiona Hyland, Wenming Xiao, Kathryn L Hunkapiller, Julie Blake, Frances Chan, Carolyn Gonzalez, Lu Zhang, Raymond R Samaha

**Affiliations:** 1 Molecular Biology Division, Applied Biosystems, Foster City, CA 94404, USA

## Abstract

**Background:**

DNA microarrays are rapidly becoming a fundamental tool in discovery-based genomic and biomedical research. However, the reliability of the microarray results is being challenged due to the existence of different technologies and non-standard methods of data analysis and interpretation. In the absence of a "gold standard"/"reference method" for the gene expression measurements, studies evaluating and comparing the performance of various microarray platforms have often yielded subjective and conflicting conclusions. To address this issue we have conducted a large scale TaqMan^® ^Gene Expression Assay based real-time PCR experiment and used this data set as the reference to evaluate the performance of two representative commercial microarray platforms.

**Results:**

In this study, we analyzed the gene expression profiles of three human tissues: brain, lung, liver and one universal human reference sample (UHR) using two representative commercial long-oligonucleotide microarray platforms: (1) Applied Biosystems Human Genome Survey Microarrays (based on single-color detection); (2) Agilent Whole Human Genome Oligo Microarrays (based on two-color detection). 1,375 genes represented by both microarray platforms and spanning a wide dynamic range in gene expression levels, were selected for TaqMan^® ^Gene Expression Assay based real-time PCR validation. For each platform, four technical replicates were performed on the same total RNA samples according to each manufacturer's standard protocols. For Agilent arrays, comparative hybridization was performed using incorporation of Cy5 for brain/lung/liver RNA and Cy3 for UHR RNA (common reference). Using the TaqMan^® ^Gene Expression Assay based real-time PCR data set as the reference set, the performance of the two microarray platforms was evaluated focusing on the following criteria: (1) Sensitivity and accuracy in detection of expression; (2) Fold change correlation with real-time PCR data in pair-wise tissues as well as in gene expression profiles determined across all tissues; (3) Sensitivity and accuracy in detection of differential expression.

**Conclusion:**

Our study provides one of the largest "reference" data set of gene expression measurements using TaqMan^® ^Gene Expression Assay based real-time PCR technology. This data set allowed us to use an alternative gene expression technology to evaluate the performance of different microarray platforms. We conclude that microarrays are indeed invaluable discovery tools with acceptable reliability for genome-wide gene expression screening, though validation of putative changes in gene expression remains advisable. Our study also characterizes the limitations of microarrays; understanding these limitations will enable researchers to more effectively evaluate microarray results in a more cautious and appropriate manner.

## Background

DNA microarray technology provides a powerful tool for characterizing gene expression on a genome scale. While the technology has been widely used in discovery-based medical and basic biological research, its direct application in clinical practice and regulatory decision-making has been questioned [1-4]. A few key issues, including the reproducibility, reliability, compatibility and standardization of microarray analysis and results, must be critically addressed before any routine usage of microarrays in clinical laboratory and regulated areas. Considerable effort has been dedicated to investigate these important issues, most of which focused on the compatibility across different microarray platforms, laboratories and analytical methods. However, in the absence of a "gold standard" or common reference for gene expression measurements, these evaluations and comparisons have often yield subjective and conflicting conclusions [5-11].

Real-time PCR is often referred to as the "gold standard" for gene expression measurements [8,12], due to its advantages in detection sensitivity, sequence specificity, large dynamic range as well as its high precision and reproducible quantitation compared to other techniques (For recent reviews, see [13-15]). The performance capabilities and ease-of-use of TaqMan based real-time PCR chemistries and instrumentation has led to widespread use of this technology as a preferred method for quantifying gene expression as well as for independent validation of microarray results [3,12,16,17]. TaqMan^® ^Gene Expression Assays (Applied Biosystems, Foster City, CA) utilize the 5' nuclease activity of AmpliTaq Gold^® ^DNA polymerase to hydrolyze a target-specific probe (TaqMan probe) bound to its target amplicon during PCR [see Additional file 1]. Each TaqMan Gene Expression Assay consists of two sequence-specific primers defining the endpoints of the amplicon and a TaqMan probe with a fluorescent reporter dye (FAM™) and a nonfluorescent quencher moiety attached to the 5' and 3' ends. Together, the primer set and the TaqMan probe provide two levels of sequence specificity. Problems associated with DNA contamination are minimized by designing primers that span at least one intron of the genomic sequence whenever possible. During each PCR extension cycle, the Taq DNA polymerase cleaves the reporter dye from the probe and once separated from the quencher. The reporter dye emits its characteristic fluorescence to allow measurement of PCR amplification as it occurs, cycle by cycle, during the highly reproducible exponential phase of PCR. This enables highly accurate and precise quantitation of gene expression over a large dynamic range.

The goal of this study is to construct a large data set based on TaqMan^® ^Gene Expression Assays and real-time PCR technology and use this data set as the reference set to objectively evaluate the gene expression measurements from two different commercial microarray platforms. The two representative commercial microarray platforms we evaluated are the Applied Biosystems Human Genome Survey Microarrays and the Agilent Human Whole Genome Oligo Microarrays. We chose these two platforms because: (1) They represent a widely used single-color array system and two-color array system, respectively; (2) both utilize long oligonucleotide probes (60mer) to achieve the best balanced sensitivity and specificity in gene expression measurements [18]; and (3) both take advantage of the latest genomic information for gene coverage and probe design, which means that the cross-mapping between the two platforms is more extensive and less ambiguous than with some other platforms. Table 1 outlines some of the key features of the two microarray platforms and TaqMan^® ^Gene Expression Assays based real-time PCR technology.

## Results

### Target selection for real-time PCR validation

In order to conduct a comprehensive and unbiased survey of the microarrays' performance, we selected the gene targets for real-time PCR validation based on the following strategies: (1) Ensure a large enough number of validation targets to provide representative overviews of the microarray performance; (2) Select genes with expression levels spanning a wide dynamic range; (3) Select genes that are represented by both microarray platforms. Validation targets were selected across the expression range from the 21,171 genes cross-mapped on both platforms – the "common genes" (See Methods for mapping methodology). Because single-color microarray systems, in general, represent more straightforward signal-abundance relationship than two-color microarray system which based on relative quantification, we used an existing Applied Biosystem data set as a reference for binning by signal. Specifically, average signals from the four technical replicates of the liver sample generated by Applied Biosystem Expression Arrays were sorted and binned into 10 bins with each bin containing equal numbers of genes. 55 genes were selected randomly from each bin yielding 550 gene targets; Another 550 targets were selected in the same fashion from data generated from the UHR sample. Finally, 275 targets were chosen randomly from differentially expressed genes (common between the Applied Biosystem data set and the Agilent data set) across the liver, lung and brain samples based on ANOVA analysis (*p *< 0.05). As a result, a total of 1375 genes were selected for real-time PCR validation using TaqMan^® ^Gene Expression Assays [see Additional file 2]. For genes with multiple TaqMan assays targeting different exon or exon-exon junctions, one assay was randomly selected without efforts in matching its location to that of corresponding probe on either microarray platform.

### Intra-platform reproducibility

**Figure 1:**
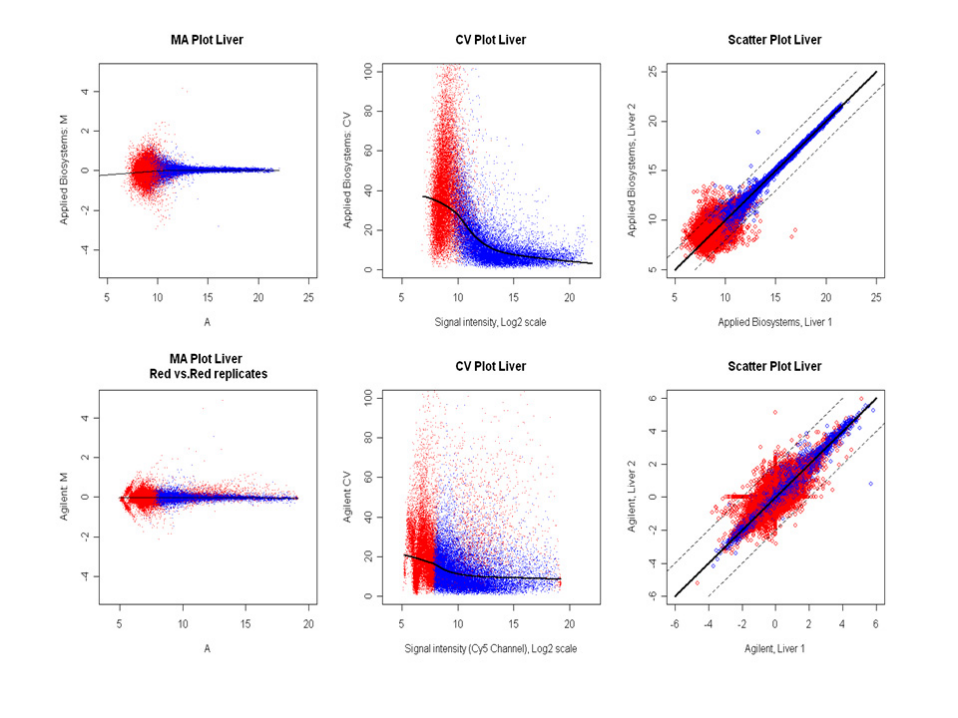
**Intra-platform reproducibility of the two microarray platforms**. Data on liver sample are shown as a representative example. All 21,171 common genes are represented in these plots. Blue points: concordantly detectable on both replicates; Red points: not detectable in either replicate. (A). M-A plots of two technical replicates analyzed by the two microarray platforms. x-axis: A = 0.5*log2 (Signal_rep1*Signal_rep2); y-axis: M = log2 (Signal_rep1/Signal_rep2). (B). Coefficients of variation (CV) for each microarray platform as a function of gene expression level across four technical replicates. For Agilent arrays, only signals of Cy5 channel were used for illustration in these plots. The black line represents a loess smoothing fitting curve to the 84,684 data points in each platform. (C). Scatter plots of the expression levels measured by each microarray platform for the two technical replicates: For Applied Biosystems arrays, expression levels are represented by signal intensity directly; for Agilent arrays, the expression levels are represented by relative ratio vs. common reference sample (UHR). The black dashed lines indicate the ± 2-fold changes.

**Figure 2:**
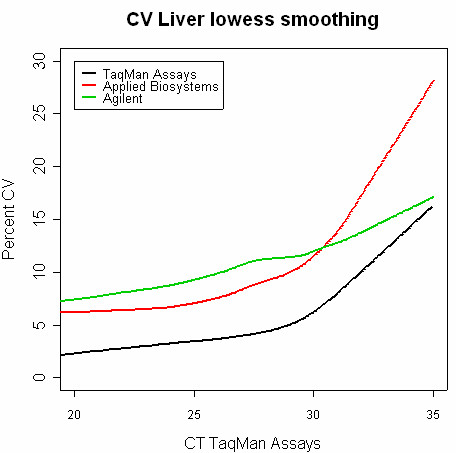
**Coefficients of variation (CV) for the two microarray platforms and TaqMan^® ^Gene Expression Assay based real-time PCR**. The CV of 1,375 genes analyzed by all three platforms is plotted as a function of gene expression level. The lines represent lowess smoothing fitting curves to the 5,500 data points in each platform.

For each microarray and real-time PCR platform, parallel gene expression data were collected on each of the four total RNA samples (human brain, lung, liver and universal reference (UHR)) in quadruplet. For the two-color Agilent arrays, comparative hybridization was performed using incorporation of Cy5 for brain/lung/liver RNA and Cy3 for UHR RNA (common reference). Using liver tissue as a representative example, the reproducibility of technical replicates for the two microarray platforms is illustrated in Figure 1. All 21,171 common genes are represented in these plots. The panel A shows the MA plots representing the pair-wise array-to-array reproducibility of each microarray platform; the panel B shows the coefficients of variation (CV) for each array platform, as a function of signal intensity, across all four technical replicates. In order to make a more direct comparison between the two microarray platforms, only red (Cy5) channel signals were used for illustrating the reproducibility of signal intensity for Agilent arrays in these plots. Because two-color systems, such as Agilent microarrays, measure relative expression level (ratio) of a given sample vs. a common reference sample, we also generated scatter plots of the expression level of two technical replicates of liver tissue (Figure 1, panel C): For the Applied Biosystems arrays, expression levels are represented as direct signal intensities; For the Agilent arrays, the expression levels are represented as relative expression level compared to the reference sample (UHR). In general, both array platforms achieve relatively good intra-platform reproducibility, in particular for the population of genes above detection threshold defined by each platform (shown in blue): For Applied Biosystems arrays, 97.4% of the detectable genes fall within 2-fold change between technical replicates; the CV range is between 6 and 22%; For Agilent arrays, 98.7% of the detectable genes fall within 2-fold change between technical replicates, the CV range is from 10 to 18%; Compared to Applied Biosystems Expression Arrays, the CV distribution of Agilent arrays appears to be less dependent on signal intensity. When the intra-platform reproducibility of microarrays was compared to that of TaqMan Gene Expression Assay based real-time PCR, using the 1375 common genes with data in the three platforms, the real-time PCR data shows significantly lower in CV across the whole dynamic range compared to the two microarray platforms (Figure 2).

### Signal detection sensitivity and accuracy

The dynamic range for most of the commercial and home brewed microarray platforms usually falls into 3–4 orders of magnitude [9,10,19], while TaqMan based real-time PCR can achieve 6–8 orders of magnitude dynamic range [20,21]. The larger dynamic range imparts TaqMan Gene Expression Assays with superior detection sensitivity (limit of detection ~ 1–5 copies per reaction [20]); we therefore used the TaqMan Gene Expression Assay data set as the reference to evaluate the performance of the two microarray platforms in terms of detection sensitivity and accuracy. First, genes that are detectable (positives: above detection threshold) and not detectable (negatives: below detection threshold) were determined for each tissue according to detection thresholds defined by each manufacturer (see Methods for detailed descriptions). We then constructed a contingency table, in which TaqMan^® ^Gene Expression Assays'present/absent calls were used as the "ground truth", and concordance with microarrays' present/absent calls were determined by calculating the True Positives, True Negatives, False Positives, and False Negatives (see Methods for detailed descriptions). Based on this matrix, the detection sensitivity and specificity for each microarray platform were determined. As listed in Table 2, the detection sensitivity and specificity, reflected by the True Positive Rate (TPR) and 1- False Positive Rate (FPR), are 76.5 % and 71.0 % for Applied Biosystems Expression Arrays, and 71.3 % and 50.0 % for Agilent arrays, respectively.

### Correlation between real-time PCR and microarrays in fold change measurements of pair-wise tissues

**Figure 3:**
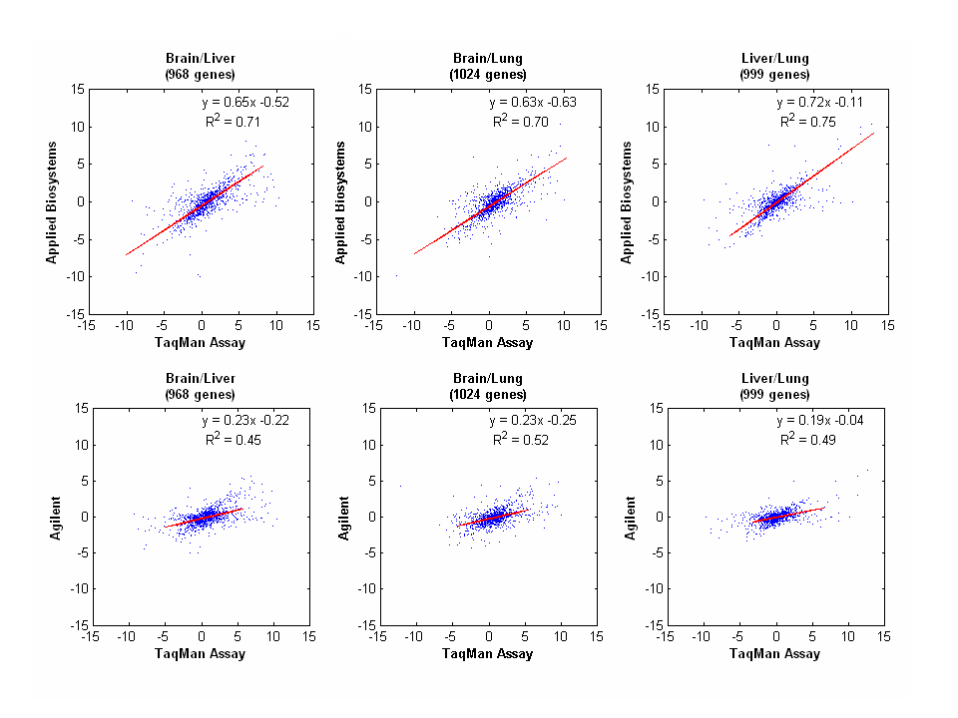
**Correlation of fold change in pair-wise tissues determined by microarray platforms and TaqMan^® ^Gene Expression Assay based real-time PCR**. y-axis, fold change determined by microarrays which is defined as: For Applied Biosystems Arrays, log2 (MedianSignal_tissue1/MedianSignal_tissue2); for Agilent arrays, log2(MedianSignal_tissue1/MedianSignal_UHR)- log2(medianSignal_tissue2/MedianSignal_UHR); x-axis, fold change determined by real-time PCR, which is defined as ΔΔCt = (Ct_tissue2-Ct_PPIA)-(Ct_tissue1-Ct_PPIA). For each pair-wise comparison, genes were filtered based on real-time PCR detection thresholds (detectable in at least 3 out of 4 technical replicates in each tissue and detectable in both tissues, the number of genes are shown in the parentheses). A robust linear regression fitting and the corresponding R^2 ^value are presented in each plot.

**Figure 4:**
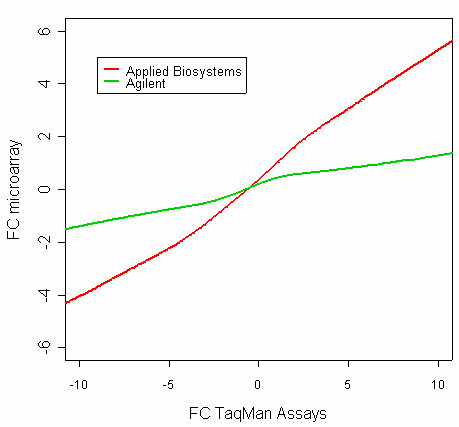
**Fold change repression in microarray platforms**. Fold change of pair-wise tissues (brain vs. liver, brain vs. lung and liver vs. lung) determined by each microarray platform (y-axis) were plotted aganinst those determined by TaqMan Assays (x-axis). Genes were filtered based on real-time PCR detection thresholds (detectable in at least 3 out of 4 technical replicates in at least one of the three tissues). The lines represent lowess smoothing fitting curves to 3,105 data points (sum of all three pair-wise tissues) in each platform.

Because different gene expression platforms utilize different technology, quantitation, and normalization methods, the absolute signal values for each platform tend to be somewhat arbitrary and not suitable for correlation analysis across different platforms. We therefore evaluated the correlation between microarrays and real-time PCR in fold change measurements between different tissues. Fold change metrics tend to cancel out systematic platform biases in absolute signal values: in addition, this is the most biologically meaningful metric. For fold change calculations, the median expression level of the four technical replicates measured for each tissue was used for each platform. For Agilent arrays, the expression level was measured as a ratio of tissue vs. UHR. For comparison of fold change between pair-wise tissues, scatter plots were generated between log2 Fold Change determined by microarrays and by TaqMan Gene Expression Assays (ΔΔCt), for every possible combination of tissue pairs, brain vs. liver, brain vs. lung and liver vs. lung, (Figure 3). Because the UHR sample was used as the reference channel in Agilent array data set, we did not include the direct fold change analysis between human tissues and UHR sample to avoid potential inconsistency caused by the two-dye bias. For each pair-wise comparison, genes were filtered based on real-time PCR detection thresholds (detectable in at least 3 out of 4 technical replicates in each tissue and detectable in both tissues). A robust linear regression fitting using bisquare weights for all data points, was performed for the scatter plot for each tissue. The Applied Biosystems data set showed better correlation with real-time PCR measurements with R^2 ^values ranging from 0.71–0.75, while the R^2 ^values for Agilent arrays range from 0.45–0.52. In addition, the slopes of the linear regression fitting curves indicated that the Applied Biosystems data set has overall less ratio compression of the extent of fold change (fitted slope 0.63–0.72) compared to the Agilent array data set (fitted slope 0.19–0.23). Fold change compression in both microarray platforms, in particular Agilent arrays, is more evident when lowess smoothing fitting was used instead of a linear regression fitting (Figure 4). The estimated range of fold changes (in log2 scale), for TaqMan Gene Expression Assays, is from -10 to 10, for Applied Biosystems arrays is -4 to 6, and for Agilent arrays, is -2 to 2, respectively.

### Correlation between real-time PCR and microarrays in expression profiles across all tissues

**Figure 5:**
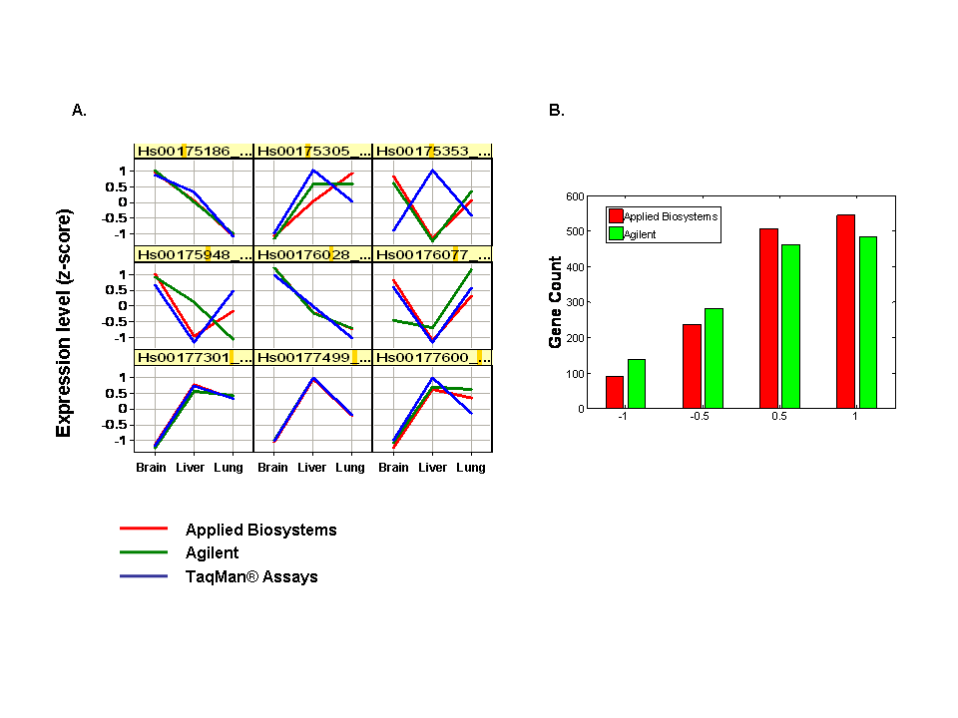
**Spearman rank-order correlation of gene expression profiles across all three tissues determined by microarray platforms and TaqMan^® ^Gene Expression Assay based real-time PCR**. (A). Example gene expression profiles on 9 genes determined by Applied Biosystems microarrays, Agilent microarrays, and TaqMan Gene Expression Assay based real-time PCR. The gene expression profile for each gene across the three tissues was determined using the median expression level of the four technical replicates followed by a z-score transformation across the three tissues for each of platforms as described in Methods. (B). Distribution of the Spearman rank-order correlation coefficients (*r*) of profiles determined by each microarray platform vs. real-time PCR.

In addition to evaluating each pair-wise combination of tissues in turn, we also evaluated the agreement in the rank order of tissues for each gene's expression profiles across all human tissues analyzed in this study (brain, liver and lung) determined by real-time PCR and microarrays. The gene expression profile for each gene across the three tissues was determined using the median expression level of the four technical replicates (Figure 5A) and rank-ordered across the three tissues. Using profiles determined by TaqMan Gene Expression Assays as the standard, the Spearman rank-order correlation coefficient (r) between each microarray platform and real-time PCR result was calculated for each gene. Because the Spearman's correlation can only attain a few different values with three data points, we separated genes into four groups, each representing a different level of agreement with the TaqMan real-time PCR reference (e.g. perfect agreement (r = 1), consistent (r = 0.5), less consistent (r = -0.5), and anti-correlation (r = -1)). As shown in Figure 5B, Applied Biosystems arrays showed 40% (544) of genes with perfect agreement and 6.3% (88) genes with anti-correlation, while Agilent arrays showed 34% (467) of genes with perfect agreement and 9.6% (132) of genes with anti-correlation. It is noteworthy that we used all the genes to calculate the correlations without filtering out genes that are not differentially expressed or not detectable across the three tissues. The inherent noise in these genes may partially account for the relatively high number of genes in the less consistent and anti-correlation classes. Another factor may potentially contribute to the anti-correlation is the difference in probe designs for microarrays and TaqMan Expression Assays. This factor will be discussed in more detail in the discussion session.

### Sensitivity and accuracy in differential expression analysis

**Figure 6:**
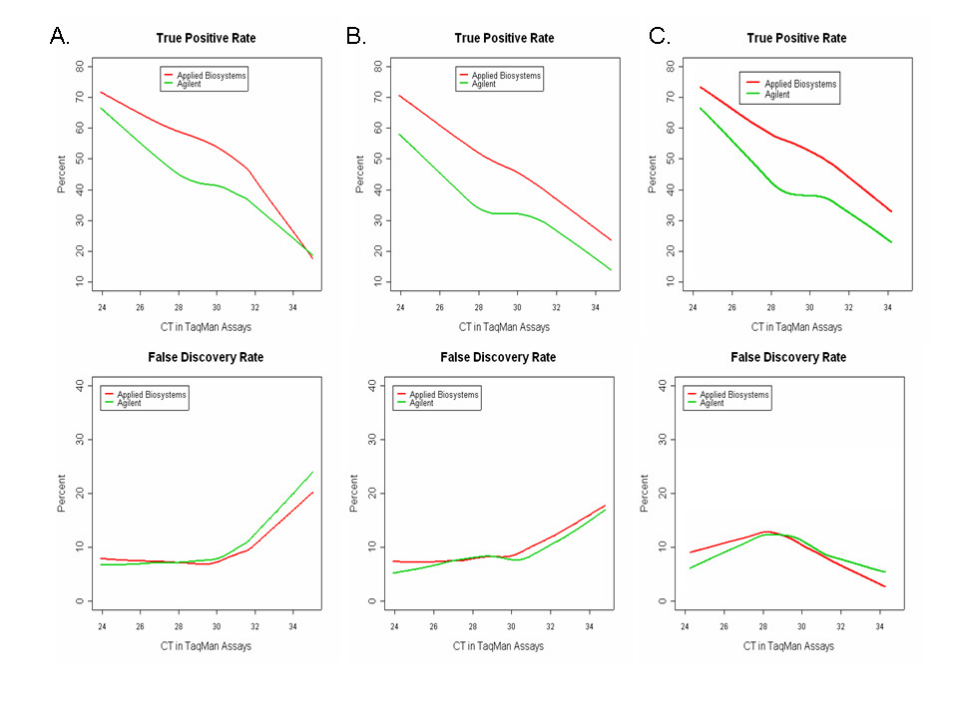
**Sensitivity and specificity in detection of differential expression at different expression levels**. For each platform, significantly differentially expressed genes for any given pair-wise tissues are determined as *p*-value < 0.05 using a student t-test (Panel A), using *p*-value adjusted according to Benjamini Horschberg multiple testing to control FDR at 5% (Panel B), or using a fold change cutoff (> 1.2-fold) for the TaqMan reference data sets while using a fold change cutoff (> 1.2-fold) and *p*-value < 0.05 based on t-test for microarray platforms (Panel C). Composite results for all three pairs of tissues (Brain vs. Liver, Brain vs. Lung, and Liver vs. Lung) were plotted. Gene expression levels are ordered according to TaqMan^® ^Gene Expression Assay measurements (average Ct between the three tissues, only genes detected in both tissues by TaqMan assays were analyzed). A sliding window containing 100 consecutive genes was constructed and moved one gene at a time to cover the whole range of Ct values. Within each sliding window, the True Positive Rate (upper panel) and False Discovery Rate (lower panel) of each microarray platform was computed and plotted as a function of gene expression level.

Finally, we evaluated the performance of the two microarray platforms in detecting differential expression using multiple statistical approaches. Because t-statistics based statistical tests take technical variations into consideration when determining true biological differences, they have become one of the most commonly applied methods to determine differentially expressed genes from microarray results. We therefore examined the sensitivity and accuracy of this approach using the TaqMan real-time PCR data as the "reference" data sets. Differentially expressed genes were determined between pair-wise tissues for both microarray and TaqMan real-time PCR reference data sets as p-value < 0.05 based on a student's t-test. To evaluate the accuracy of using t-test to define the "true" differential expressions in the reference set, we performed power calculation (estimate of type II errors of t-test, e.g. false negatives) on the TaqMan reference data sets. Our results showed that, with four technical replicates, 100% power can be achieved to detect a 1.5-fold change, and at least 90% power can be achieved to detect a 1.2-fold change with p-value < 0.05 in the TaqMan reference data sets [see Additional file 7). To control for type I errors (false positives), t-statistics was performed with or without a 5% FDR correction (p-value adjusted by Benjamini and Hochberg False Discovery Rate multiple testing corrections) and the results are shown in Figure 6 (Panel A&B). To dissect the accuracy of microarrays in detecting differentially expressed genes at different expression levels, we plotted the corresponding TPR (sensitivity) and FDR of each microarray platform as a function of the average expression levels for the three tissues. As shown in Figure 6 (Panel A&B), at the highest expression level, both microarray platforms displayed reasonably good sensitivities (upper panels): 70–72% TPR for Applied Biosystems arrays and 58–65% TPR for Agilent arrays; the performance drops as the expression level decreases, and at the lowest expression level, the TPR is 20–30% for Applied Biosystems arrays and 15–20% for Agilent arrays. A more dramatic change can be seen for the FDR plots (bottom panels). For both microarray platforms, a relative constant level of false findings (FDR 6–7%) was observed for genes with high and medium expression levels (Ct < 30), after which FDR almost doubles (Figure 6, Panel B, with FDR control) and triples (Figure 6, Panel A, without FDR control) for genes at low expression levels. Another approach is to forgo using a t-test to define differently expressed genes with the TaqMan data; but considering it the reference or gold standard data use the unfiltered fold change as the measure of differential expression. We therefore defined the differentially expressed genes in the TaqMan data set as genes with an average fold change > 1.2 between pair-wise tissues, while for microarray data sets, the differently expressed genes were defined as p-value < 0.05 and average fold change > 1.2 between pair-wise tissues. As shown in Figure 6, Panel C, while the distribution of the true positive rates for both microarray platforms were similar as the ones observed previously, the true positive rates appeared to improve slightly especially for genes with lower expression levels. Interestingly, the false discovery rates also appeared to drop slightly as the expression level decreased. This result may be explained by the different criteria used to define the differentially expressed genes in the "reference" data sets and in the microarray data sets. Since a t-test was used for microarray data sets on top of a fold change cutoff to define differentially expressed genes, fewer genes with lower expression levels and therefore higher variability will pass the t-test cutoffs. On the other hand, because the TaqMan reference data sets used a constant fold change cutoff without a t-test to define differentially expressed genes, more low expressed genes will potentially pass this cutoff (Figure 3). As a result, the true positive rate for the microarray platforms appears to increase while the false discovery rate shows a decreasing trend for genes at the low expression levels.

**Figure 7:**
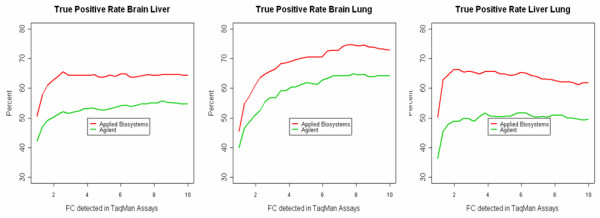
**Sensitivity in detection of differential expression for different fold changes**. For each platform, significantly differentially expressed genes for any given pair-wise tissues are determined using t-test at 95% significance level (*p*-value = 0.05). Using one-sample z-test, genes showing "at least F fold change" with 95% confidence are grouped based on TaqMan^® ^Gene Expression Assays data set. True Positive Rates of each microarray platform was plotted as a function of Fold Change cut-off (range from 1.2 – 10) for each pair-wise tissues.

Another interesting measure of microarrays performance is their sensitivity in detecting relatively low fold changes. To address this question, we determined the differentially expressed genes as described above and plotted the TPR for each microarray platform as a function of the fold changes determined by the real-time PCR data. As shown in Figure 7, the sensitivity in detecting lower fold changes (1.2–2 fold) is dramatically lower for both microarray platforms: with TPR 50–65% for Applied Biosystems arrays and TPR 40–55% for Agilent arrays; the sensitivity becomes relatively stable at higher fold changes (> 2-fold) (Figure 7).

**Figure 8:**
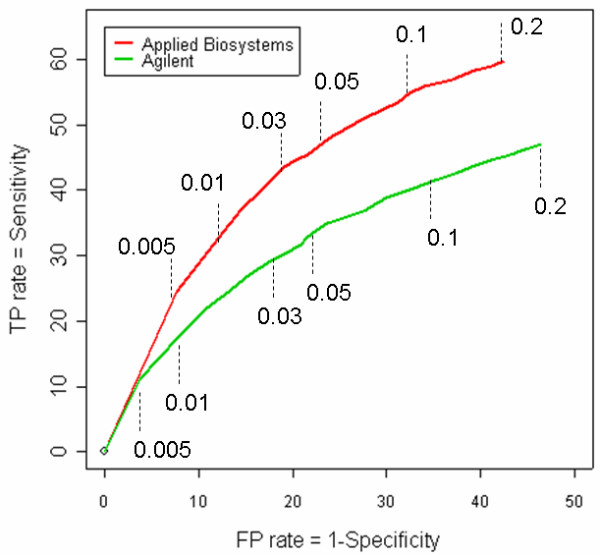
**ROC curve for accuracy in detection of differential expression at different FDR thresholds**. Significantly differentially expressed genes are defined as p < 0.05 in student t-test using TaqMan Gene Expression data set as a reference. For each microarray platform, on top of the p-value criteria (p < 0.05 in student t-test), a series of FDR (0–20%) were also applied to achieve increasing stringency. Each point on the ROC curve of a given microarray platform represents the sensitivity (true positive rate) and 1- specificity (false positive rate) at a given FDR level (labeled on dashed lines).

Multiple testing with FDR adjustments helps to reduce false positives in t-statistics and has been widely applied in differential expression analysis of microarray data. To investigate the overall accuracy of microarrays in detecting differential expression at different FDR levels, a Receiver Operating Characteristics (ROC) curve was constructed for each microarray platform (Figure 8). In this graph, a series of FDR levels (0–20%) was applied to microarray data sets, while significance threshold was kept at *p *< 0.05 for TaqMan real-time platform. At each FDR level indicated by the dashed lines, the corresponding True Positive Rate (sensitivity) was plotted against the False Positive Rate (1- specificity). As shown in Figure 8, when the stringency was increased for microarray platforms with increased FDR (0–20%), the sensitivity of microarrays dramatically increased from 0% to 60% for Applied Biosystems arrays and from 0% to 47% for Agilent arrays, however, the specificity of both arrays dropped in the mean time. The false positive rate increased from 0% to 42% for Applied Biosystems arrays and increased from 0% to 47% for Agilent arrays, respectively.

#### GEO accession

The complete data sets of this study can be accessed from Gene Expression Omnibus (GEO) [28], accession ID GSE4214.

## Discussion

Although microarrays have been extensively used as discovery tools for biological and biomedical studies, the challenge remains whether this technology can be reliably applied in clinical practice and regulatory decision making, where high precision and accuracy in performance are required. A series of studies have been reported on evaluating performance across various commercial and home-brewed microarray platforms, however, most of these studies focused on evaluating the level of concordance across different microarray platforms. While these analyses emphasized critical issues such as the compatibility across different microarray platforms, they tended to result in conflicting conclusions because the "relative to relative" nature of such approaches. What is lacking in these studies is a "gold standard" data set that allows an evaluation of different microarray platforms based on a common "ground truth". One commonly used approach for setting up such "ground truth" is by spiking in bacterial synthetic transcripts with known concentrations in series of dilutions over a large dynamic range [22], however, the limitation of this approach is that the information is asserted from very limited transcripts, and it is also very prone to experimental artifacts. An alternative strategy to set up the "ground truth" is using a well accepted reference data set generated by a reliable independent technology, such as real-time PCR for gene expression measurements. In this study, we have constructed a large reference data set of gene expression measurements using TaqMan Gene Expression Assays and real-time PCR technology. We also demonstrated how to use such a data set to evaluate the performance of different microarray platforms.

We first evaluated the detection sensitivity and accuracy of the two selected microarray platforms using TaqMan Gene Expression Assays and real-time PCR data set as the reference. We chose to use the detection thresholds that are recommended by each manufacturer as the base line for comparison. These recommended thresholds are somewhat arbitrary and are not necessarily based on the same parameters, nevertheless, these detection thresholds are widely adopted by researchers and therefore evaluating their effect on detection sensitivity and accuracy can prove useful in further refining them and better interpreting microarray results. Our results showed that both of the microarray platforms can achieve reasonably good sensitivity in signal detection, while the specificity tends to be relatively low, especially for Agilent microarrays, with a ~ 50% false positive rate. It is worth noting that the differences in detection sensitivity and specificity we observed could be caused by less optimal bioinformatics/algorithms used to define the detection thresholds and do not necessarily reflect the inherent qualities or accuracies of the respective platforms. Several strategies could be developed to improve the detection specificity of microarrays, including improving probe design, hybridization conditions which would minimize the effects of cross-hybridization, as well as improving image analysis software/algorithms to facilitate more accurate signal quantification and detection thresh-holding.

This study also evaluated correlation in detecting differential expression between microarray platforms and TaqMan real-time PCR platform (Figure 3 and Figure 5). Our analysis also provided a high-resolution examination of the performance of microarrays in detecting differential expression at different expression levels as well as at different fold changes. We validated that microarrays have acceptable sensitivity and accuracy in detecting differential expression, especially for genes with high and medium expression levels and for detecting > 2-fold changes. These results support the notion that microarrays, as exploratory tools for genome-wide gene expression screening, can achieve acceptable reliability in performance.

Our study also characterized some of the limitations of microarrays, in particular the ratio compression phenomena as shown in Figure 4. A certain level of fold change compression is expected for microarray platforms due to various technical limitations, including limited dynamic range, signal saturations, and cross-hybridizations. The two-color system analyzed in this study (Agilent microarrays), appears to have more severe ratio compression, which could be attributed to several factors: (1) The concentration of the 60mer probes on Agilent microarrays depends on the coupling efficiency of the in-situ oligonucleotide synthesis, and on probe length. Lower efficiency may result in low probe concentration and therefore limit the dynamic range of the platform; (2) Two-color systems such as Agilent arrays, utilize two different fluorescent dyes that have different dynamic ranges and quantum yields. These intrinsic differences may be partially adjusted by intra-array intensity-dependent normalization but may not be completely eliminated. Theoretically, dye swapping experiments may help to further adjust these biases introduced by two different dyes. In reality, however, dye-swapping is not always practical due to cost and limitations in sample amount. Finally, ratio compression can be also introduced by certain data-processing/normalization algorithms that aim to reduce variances (e.g. lowess normalization for Agilent microarrays and RMA method for Affymetrix microarrays). Our analysis suggests that the optimal balance between the two parameters will eventually determine the overall accuracy in detection of differential expression, for a given microarray platform. Other microarray limitations revealed by our study include the significant decrease in overall accuracy of differential expression detection at low expression level (Figure 6) and the relatively poor sensitivity in detecting small fold changes (i.e. < 2-fold). Although these limitations have been previously suspected by many, the large scale "reference" data set provided by our study provides a more quantitative view of these limitations for the first time.

Lastly, it is noteworthy that although TaqMan Gene Expression Assay based real-time PCR is a well accepted "gold standard" for gene expression measurements, we are aware that it has its own limitations and is also affected by experimental errors. In addition, different strategies in probe designs for microarrays (usually 3' biased and targeting a composite of transcripts) and TaqMan Gene Expression Assays (usually without *a priori *bias and targeting a single or subset of transcripts) may also account for a small percentage of the discordance observed between the microarrays and real-time PCR results. For example, the gene expression profiles of gene NM_003640 measured by both microarray platforms are highly correlated with each other but anti-correlates with the profile measured by TaqMan Gene Expression Assay (Hs00175353_m1, Figure 5A). NM_003640 is a relative long transcript with 5917 bases and 37 exons. While the TaqMan assay was designed against the exon 2 and exon 3 junctions, which is > 4 kb from the 3' end, the microarray probes were usually designed close to 3' end (mostly within 1.5 kb from 3' end for Applied Biosystems probes). In this particular instance, the data suggest that the TaqMan gene expression assay is potentially detecting additional splice variants than the array probes. This difference in probe designs may result in quantifying different population of transcripts (e.g. product of alternative splicing or degradation) by microarrays or TaqMan assays. These factors may change the absolute metrics (i.e. TRP, TFP, and anti-correlation rate); nevertheless, they would not change the general conclusions and trends we observed. We think that most of the discrepancies between TaqMan based real-time PCR and microarrays are due to the sensitivity limits of a PCR based approach vs. a hybridization based approach. It is clear that at high expression levels, there is a much better correlation between the two approaches (Figure 6). These factors may change the absolute metrics (i.e. TRP, TFP, and anti-correlation rate); nevertheless, they would not change the general conclusions and trends we observed. We think that most of the discrepancies between TaqMan based real-time PCR and microarrays are due to the sensitivity limits of a PCR based approach vs. a hybridization based approach. It is clear that at high expression levels, there is a much better correlation between the two approaches (Figure 6).

## Conclusion

Our study provides one of the largest "reference" data set of gene expression measurements using TaqMan^® ^Gene Expression Assay based real-time PCR technology. We also provide novel analysis approaches for evaluating different micorarray platforms as well as performing cross-platform correlations. As a result of this study, we recommend using "reference" data sets generated by real-time PCR to evaluate critical aspects of microarray platforms, including signal detection threshold, fold change correlation between pair-wise tissues, profile correlation across multiple tissues, as well as sensitivity and specificity in signal detection and differential expression. We conclude that microarrays are invaluable discovery tools with acceptable reliability for genome-wide gene expression screening. Understanding the limitations of microarrays characterized by our study will help us to better apply this technology and interpret its results more cautiously.

## Methods

### RNA samples

Total RNA samples of human whole brain (# 929565), liver (# 929564), lung (#929566), and the universal human reference sample (UHR, # 929563) were purchased from Stratagene (La Jolla, CA). The quality and integrity of the total RNA was evaluated on the 2100 Bioanalyzer (Agilent Technologies, Palo Alto, CA), and the same samples were divided into individual aliquots for the gene expression analysis on the two different microarray platforms and for the TaqMan Gene Expression Assay based real-time PCR analysis.

### Applied Biosystems Expression Array analysis

The Applied Biosystems Human Genome Survey Microarray (P/N 4337467) contains 31,700 60-mer oligonucleotide probes representing 27,868 individual human genes. Digoxigenin-UTP labeled cRNA was generated and amplified from 1 *μ*g of total RNA from each sample using Applied Biosystems Chemiluminescent RT-IVT Labeling Kit v 1.0 (P/N 4340472) according to the manufacturer's protocol (P/N 4339629). Array hybridization was performed for 16 hrs at 55°C. Chemiluminescence detection, image acquisition and analysis were performed using Applied Biosystems Chemiluminescence Detection Kit (P/N 4342142) and Applied Biosystems 1700 Chemiluminescent Microarray Analyzer (P/N 4338036) following the manufacturer's protocol (P/N 4339629). Images were auto-gridded and the chemiluminescent signals were quantified, background subtracted, and finally, spot- and spatially-normalized using the Applied Biosystems 1700 Chemiluminescent Microarray Analyzer software v 1.1 (P/N 4336391). Four technical replicates were performed on each sample, a total of 16 microarrays were used for the analysis. For inter-array normalization, a global median normalization was applied across all microarrays to achieve the same median signal intensities for each array. Besides using the default global median normalization method, we also investigated several other normalization methods for the Applied Biosystems data set, including Quantile normalization [23], scale normalization [24] and Variance Stabilization Normalization (VSN) [25], using the limma and vsn packages of R/Bioconductor [29]. Marginal difference was observed in outcomes among different normalization methods, including the intra-platform reproducibility [see Additional file 5] and fold change correlation with TaqMan assays [see Additional file 6].

### Agilent Whole Human Genome Oligo Microarray analysis

The Agilent Whole Human Genome Oligo Microarray (G4112A) contains 44,000 60-mer oligonucleotide probes representing 41,000 unique genes and transcripts. Probe labeling and hybridization were carried out following the manufacturer's specified protocols. Briefly, amplification and labeling of 5 ug of total RNA was performed using Cy5 for brain/liver/lung RNA and Cy3 for the reference RNA (Stratagene UHR). Hybridization was performed for 16 hrs at 60°C and arrays were scanned on an Agilent DNA microarray scanner. Following the manufacturer's protocol, Agilent's Stabilization and Drying Solution (#5185–5979) was used to protect against the ozone-induced degradation of cyanine dyes on microarray slides during hybridization and processing steps. Images were analyzed and data were extracted, background subtracted and normalized using the standard procedures of Agilent Feature Extraction Software A.7.5.1. Four technical replicates were performed for each pair of RNA samples (brain vs. UHR, liver vs. UHR, and lung vs. UHR), a total of 12 arrays were analyzed. Linear & LOWESS, which is the default normalization method in the Agilent Feature Extraction Software A.7.5.1, were applied for normalizing Agilent microarrays. This method does a linear normalization across the entire range of data, and then applies a non-linear normalization (LOWESS) to the linearized data set.

### TaqMan^® ^Gene Expression Assay based real-time PCR

Expression of mRNA for 1375 genes was measured in each of the three human tissues and the UHR total RNA samples by real-time PCR using TaqMan^® ^Gene Expression Assays on ABI PRISM 7900 HT Sequence Detection System (Applied Biosystems, Foster City, CA). ~ 5 ug of total RNA of each sample was used to generate cDNA using the ABI High Capacity cDNA Archiving Kit (Applied Biosystems, Foster City, CA) and the real-time PCR reactions were carried out following the manufacturer's protocol. TaqMan^® ^Gene Expression Assay IDs are listed [see Additional file 3]. Four technical replicates were run for each gene in each sample in a 384-well format plate and a total of 64 plates (16 plates for each sample) were run in this study. On each plate, three endogenous control genes (RPS18, PPIA (Alias: cyclophilin A) and GAPDH) and one no-template-controls (NTC) were also run in quadruplicates. We chose PPIA for normalization across different genes based on that this gene showed the most relatively constant expression in different tissue samples (data not shown).

### Cross-mapping between microarray platforms

For a direct comparison of the Applied Biosystems Human Whole Genome Survey Microarrays and the Agilent Whole Human Whole Genome Oligo Microarrays, we identified a set of genes represented on both platforms. The cross-mapping was done by using BLAST to compare Applied Biosystems 60 mer probe sequences to the target transcript sequences interrogated by the probes on Agilent arrays (GEO [28] platform GPL1708) and only probes with 100% sequence identity were included in the final gene set. When multiple probes from one platform were mapped to one probe from the other platform, a one to one probe pair was randomly selected. As a result, 21171 common genes represented by both platforms were identified [see Additional file 4].

### Data analysis

Statistical analyses were performed with the software packages MATLAB^® ^(Mathworks, Natick, MA), R/Bioconductor [29], and Spotfire Functional Genomic (Spotfire, Göteborg, Sweden).

### Signal detection analysis

Detection thresholds are defined according to each platform manufacturer's recommendation. For TaqMan Gene Expression Assays, detection threshold is set as Ct < 35 and Stdev (of 4 technical replicates) < 0.5; for Applied Biosystems Expression Arrays, detection threshold is set as Signal to Noise ratio (S/N) > 3 and quality flag < 5000; for Agilent arrays, detection threshold is set based on multiple parameters, including (1) WellAboveBackground (Signal/Background > 3.0); (2) Positive&Significant vs. Background (*p *< 0.01); and (3) they are not saturated, non-Uniform or population outliers in signals of feature and background. Detection in each tissue was defined as detectable in 3 out of 4 technical replicates within each platform. Using TaqMan^® ^Gene Expression Assays calls as the reference, contingency tables were constructed against microarray platforms, in which True Positives (TP, detectable by both TaqMan Assay and Microarray), True Negative (TN, not detectable by either TaqMan Assay nor Microarray), False Positive (FP, detectable by Microarrays but not by TaqMan Assays), and False Negative (FN, detectable by TaqMan Assays but not by Microarrays). Based on this matrix, the following statistics were calculated for each microarray platform [26]:

True positive rate, TPR=TPTP+FN
 MathType@MTEF@5@5@+=feaafiart1ev1aaatCvAUfKttLearuWrP9MDH5MBPbIqV92AaeXatLxBI9gBaebbnrfifHhDYfgasaacH8akY=wiFfYdH8Gipec8Eeeu0xXdbba9frFj0=OqFfea0dXdd9vqai=hGuQ8kuc9pgc9s8qqaq=dirpe0xb9q8qiLsFr0=vr0=vr0dc8meaabaqaciaacaGaaeqabaqabeGadaaakeaacqqGubavcqqGqbaucqqGsbGucqGH9aqpdaWcaaqaaiabdsfaujabdcfaqbqaaiabdsfaujabdcfaqjabgUcaRiabdAeagjabd6eaobaaaaa@3913@

False positive rate, FPR=FPFP+TN
 MathType@MTEF@5@5@+=feaafiart1ev1aaatCvAUfKttLearuWrP9MDH5MBPbIqV92AaeXatLxBI9gBaebbnrfifHhDYfgasaacH8akY=wiFfYdH8Gipec8Eeeu0xXdbba9frFj0=OqFfea0dXdd9vqai=hGuQ8kuc9pgc9s8qqaq=dirpe0xb9q8qiLsFr0=vr0=vr0dc8meaabaqaciaacaGaaeqabaqabeGadaaakeaacqqGgbGrcqqGqbaucqqGsbGucqGH9aqpdaWcaaqaaiabdAeagjabdcfaqbqaaiabdAeagjabdcfaqjabgUcaRiabdsfaujabd6eaobaaaaa@38DB@

False discovery rate, FDR=FPTP+FP
 MathType@MTEF@5@5@+=feaafiart1ev1aaatCvAUfKttLearuWrP9MDH5MBPbIqV92AaeXatLxBI9gBaebbnrfifHhDYfgasaacH8akY=wiFfYdH8Gipec8Eeeu0xXdbba9frFj0=OqFfea0dXdd9vqai=hGuQ8kuc9pgc9s8qqaq=dirpe0xb9q8qiLsFr0=vr0=vr0dc8meaabaqaciaacaGaaeqabaqabeGadaaakeaacqqGgbGrcqqGebarcqqGsbGucqGH9aqpdaWcaaqaaiabdAeagjabdcfaqbqaaiabdsfaujabdcfaqjabgUcaRiabdAeagjabdcfaqbaaaaa@38C7@

### Gene expression profile correlation between microarray and TaqMan^® ^Gene Expression Assays

To make a more direct comparison on gene expression profiles determined by single-color and two-color platforms, data from Applied Biosystems arrays and TaqMan Assays were transformed using UHR sample as a reference to generate brain vs. UHR, liver vs. UHR and lung vs. UHR ratios. For each individual gene, a median gene expression profile across the three tissue samples (brain, liver, and lung) was determined z-score transformed across the three tissues: Zx=X−μ¯σ
 MathType@MTEF@5@5@+=feaafiart1ev1aaatCvAUfKttLearuWrP9MDH5MBPbIqV92AaeXatLxBI9gBaebbnrfifHhDYfgasaacH8akY=wiFfYdH8Gipec8Eeeu0xXdbba9frFj0=OqFfea0dXdd9vqai=hGuQ8kuc9pgc9s8qqaq=dirpe0xb9q8qiLsFr0=vr0=vr0dc8meaabaqaciaacaGaaeqabaqabeGadaaakeaacqqGAbGwcqWG4baEcqGH9aqpdaWcaaqaaiabdIfayjabgkHiTGGaciqb=X7aTzaaraaabaGae83Wdmhaaaaa@362F@, where *X *is the median expression level (tissue vs. UHR ratio) of the four technical replicates for a given tissue and given gene, μ¯
 MathType@MTEF@5@5@+=feaafiart1ev1aaatCvAUfKttLearuWrP9MDH5MBPbIqV92AaeXatLxBI9gBaebbnrfifHhDYfgasaacH8akY=wiFfYdH8Gipec8Eeeu0xXdbba9frFj0=OqFfea0dXdd9vqai=hGuQ8kuc9pgc9s8qqaq=dirpe0xb9q8qiLsFr0=vr0=vr0dc8meaabaqaciaacaGaaeqabaqabeGadaaakeaaiiGacuWF8oqBgaqeaaaa@2E81@ is the average expression level across all three tissues for this gene, and *σ *is the standard deviation of gene expression level across all three tissues for this gene. Using the profile determined by TaqMan^® ^Gene Expression Assays as the reference, a Spearman rank-order correlation coefficient MATLAB^® ^(Mathworks, Natick, MA) was calculated against this reference for each of the two microarray platforms.

### Power calculation

To calculate the power for the TaqMan real-time PCR platform for genes with different expression levels, Ct values of all assays in the three tissues were sorted and partitioned into four bins with equal numbers of assays. These bins span Ct intervals [10, 26.8], [26.9, 28.4], [28.5, 30.4] and [30.5, 35] and represent genes with high, medium high, medium low and low different expression levels, respectively. Average standard deviation was calculated for each bin and their power to detect different fold changes with *p*-value < 0.05 or with *p*-value < 0.001 (equivalent of FDR < 5%) using four technical replicates was calculated based on methods described previously [27].

### Differential expression analysis

Significantly differentially expressed genes between different pairs of tissues were defined as *p*-value < 0.05 based on a student's t-test. Using calls from TaqMan^® ^Gene Expression Assays as the reference, contingency tables were constructed against microarray platforms, in which the concordance between microarray platforms and the TaqMan^® ^Gene Expression Assays was determined taking into considerations of both *p*-value significance and fold change directions (up or down regulation). Specifically, True Positives (TP, *p *< 0.05 for both TaqMan Assay and Microarray and fold change in the same direction), True Negative (TN, *p *> 0.05 for both TaqMan Assay and Microarray), False Positive (FP, *p *> 0.05 for TaqMan Assay and *p *< 0.05 for Microarray, or *p *< 0.05 for both TaqMan Assay and Microarray and fold change in opposite direction), and False Negative (FN, *p *< 0.05 for TaqMan Assay and *p *> 0.05 for Microarray). Based on this matrix, the TPR, FPR, FDR and accuracy were calculated for each microarray platform as described above [26]. Similar analysis were performed using different criteria for determining differentially expressed genes, including using t-test with FDR correction at 5% (*p*-value adjusted by Benjamini and Hochberg False Discovery Rate multiple testing corrections) in both microarray and TaqMan reference data sets, or using a fold change cutoff (> 1.2 fold) in TaqMan reference data sets while using the same fold change cutoff and a t-test (*p*-value < 0.05) in microarray data sets.

## Authors' contributions

YW conceived and designed the study, participated in the microarray data collection, performed data analysis and wrote the article. CB performed statistical analysis and participated in writing the article. FH participated in the design of the study and data analysis. WX carried out the cross-platform gene mapping. KLH and JB participated in the real-time PCR data collection. FC, CG and LZ participated in the microarray data collection. RRS contributed to conception and design of the study, and to revising and writing of the article.

## Supplementary Material

Additional File 1 **Scheme of TaqMan^® ^Gene Expression Assay Based Real-time PCR**. A TaqMan probe is designed to anneal to the target sequence between the traditional forward and reverse PCR primers. A fluorescent reporter dye and a quencher moiety are attached to the 5' and 3' ends of this TaqMan probe and when the probe is intact, the reporter dye emission is quenched. During each PCR extension cycle, the AmpliTaq Gold^® ^DNA polymerase has an intrinsic 5' to 3' nuclease activity and cleaves the reporter dye from the probe. Once separated from the quencher, the reporter dye emits its characteristic fluorescence. Because the amount of fluorescent signal released during each cycle of amplification is proportional to the amount of product generated, this provides the basis for the quantitative measurements of gene expressions. Click here for file

Additional File 2 **1375 gene targets for TaqMan Assay validation were selected to span a wide dynamic range in expression level**. Scatter plots between two technical replicates for liver and UHR samples were shown for the 21,171 common genes (shown in light grey) for each microarray platform. The 1375 gene targets (blue points) spanning wide dynamic range of expression levels were selected for TaqMan Assay validation. Click here for file

Additional File 3 This file contains a gene list of 1375 genes with their corresponding TaqMan^® ^Gene Expression Assay IDs, Applied Biosystem Human Genome Survey Microarray Probe IDs, and Agilent Human Whole Genome Oligo Microarray Probe IDs. Click here for file

Additional File 4 This file contains the list of 21171 common genes represented by Applied Biosystems Human Genome Survey Microarrays and Agilent Human Whole Genome Oligo Microarrays. Click here for file

Additional File 5 **Comparison of different normalization methods: Coefficients of variation (CV) of technical replicates of Applied Biosystems microarrays**. Data on liver sample are shown [8,12]as a representative example. All 33,096 features are represented in this plot. Coefficients of variation (CV) across four technical replicates were calculated using data normalized by different normalization methods, and plotted as a function of average gene expression level. Different normalization methods showed little difference in CV distribution across technical replicates. Click here for file

Additional File 6 **Comparison of different normalization methods: Correlation of fold change in pair-wise tissues determined by Applied Biosystems microarrays and TaqMan^® ^Gene Expression Assay based real-time PCR**. Data on Liver vs. Lung samples are shown as a representative example. y-axis, fold change determined by microarrays which is defined as: log2 (MedianSignal_tissue1/MedianSignal_tissue2); x-axis, fold change determined by real-time PCR, which is defined as ΔΔCt = (Ct_tissue2-Ct_PPIA)-(Ct_tissue1-Ct_PPIA). Genes were filtered based on real-time PCR detection thresholds (detectable in at least 3 out of 4 technical replicates in each tissue and detectable in both tissues, the number of genes are shown in the parentheses). A robust linear regression fitting and the corresponding R^2 ^value are presented in each plot. Different normalization methods showed little difference in fold change correlation with TaqMan assays. Click here for file

Additional File 7 **Power calculation for the TaqMan reference data sets**. The power of the TaqMan real-time PCR platform to detect different fold changes using four technical replicates was calculated as described in the Methods for genes with different expression levels. (A). with *p*-value < 0.05 ; (B). With *p*-value < 0.001 (equivalent of FDR < 5%). Click here for file

## Figures and Tables

**Table 1:** Overview of the two microarray platforms and TaqMan^® ^GeneExpression Assay based real-time PCR

	**Applied Biosystems**	**Agilent**	**TaqMan Gene Expression Assays**
**Platforms**	Human Genome Survey Microarray	Human Whole Genome Arrays	TaqMan Expression Assays
**Technology**	Hybridization	Comparative Hybridization	5' Nuclease Chemistry & Real Time PCR
**Probe (bases)**	60mer	60mer	TaqMan Primer & Probes
**Substrate**	Nylon	Glass slide	-
**Deposition**	Contact Spotting	In-situ Ink Jet Printing	-
**Detection**	One-color Chemiluminescence	Two-color Cy3/Cy5 Fluorescence	One-color FAM Fluorescence
**Software**	1700 Chemiluminescence Microaray Analyzer Software v 1.1	Feature Extraction A 7.5.1	SDS 2.1
**Total Probes**	33,096 probes	44,000 probes	1375 Selected Targets

**Table 2:** Sensitivity and accuracy in signal detection. TaqMan^® ^Gene Expression Assays calls were used as the "ground truth" to calculate the True Positive, True Negative, False Positive, False Negative, True Positive Rate (TPR) and False Positive Rate (FPR) as described in Methods. Detection thresholds for each platform were defined according to corresponding manufacturer's recommendations and described in Methods. Results are shown as sum of the Brain, Liver and Lung tissues

**Platforms**	**True Positive**	**True Negative**	**False Positive**	**False Negative**	**Sensitivity (TPR)**	**Specificity (1- FPR)**
**TaqMan^® ^Gene Expression Assays**	3286	839	0	0		
**Applied Biosystems**	2511	591	248	775	76.5%	71.0
**Agilent**	2345	437	402	941	71.3%	50.0%
