# Supplementary figures and images for: Large scale real-time PCR validation on gene expression measurements from two commercial long-oligonucleotide microarrays
Source: BMC Genomics. 2006 Mar 21;7:59. doi: 10.1186/1471-2164-7-59 (PMC1435885; doi:10.1186/1471-2164-7-59)

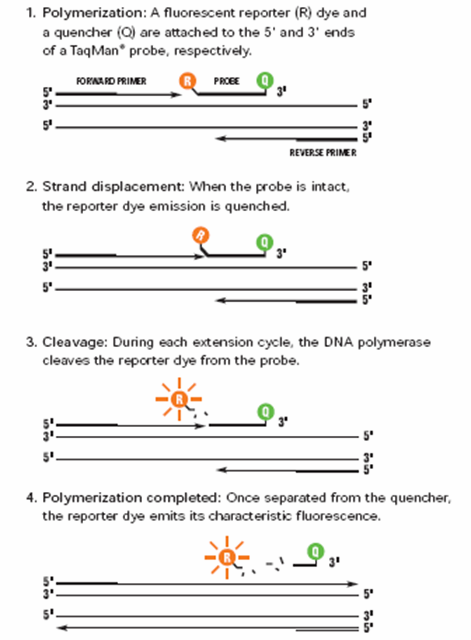

Supplement: Additional File 1 — Scheme of TaqMan® Gene Expression Assay Based Real-time PCR. A TaqMan probe is designed to anneal to the target sequence between the traditional forward and reverse PCR primers. A fluorescent reporter dye and a quencher moiety are attached to the 5' and 3' ends of this TaqMan probe and when the probe is intact, the reporter dye emission is quenched. During each PCR extension cycle, the AmpliTaq Gold® DNA polymerase has an intrinsic 5' to 3' nuclease activity and cleaves the reporter dye from the probe. Once separated from the quencher, the reporter dye emits its characteristic fluorescence. Because the amount of fluorescent signal released during each cycle of amplification is proportional to the amount of product generated, this provides the basis for the quantitative measurements of gene expressions. [file 1471-2164-7-59-S1.png]

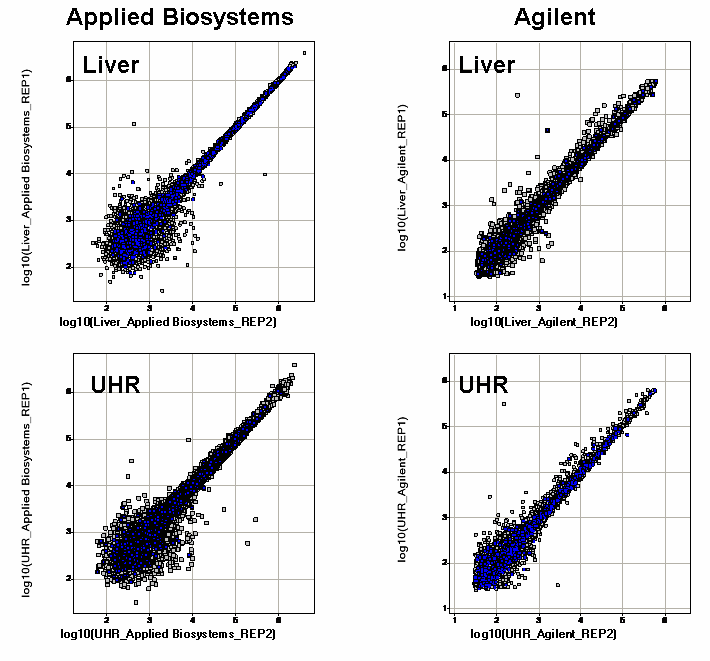

Supplement: Additional File 2 — 1375 gene targets for TaqMan Assay validation were selected to span a wide dynamic range in expression level. Scatter plots between two technical replicates for liver and UHR samples were shown for the 21,171 common genes (shown in light grey) for each microarray platform. The 1375 gene targets (blue points) spanning wide dynamic range of expression levels were selected for TaqMan Assay validation. [file 1471-2164-7-59-S2.png]

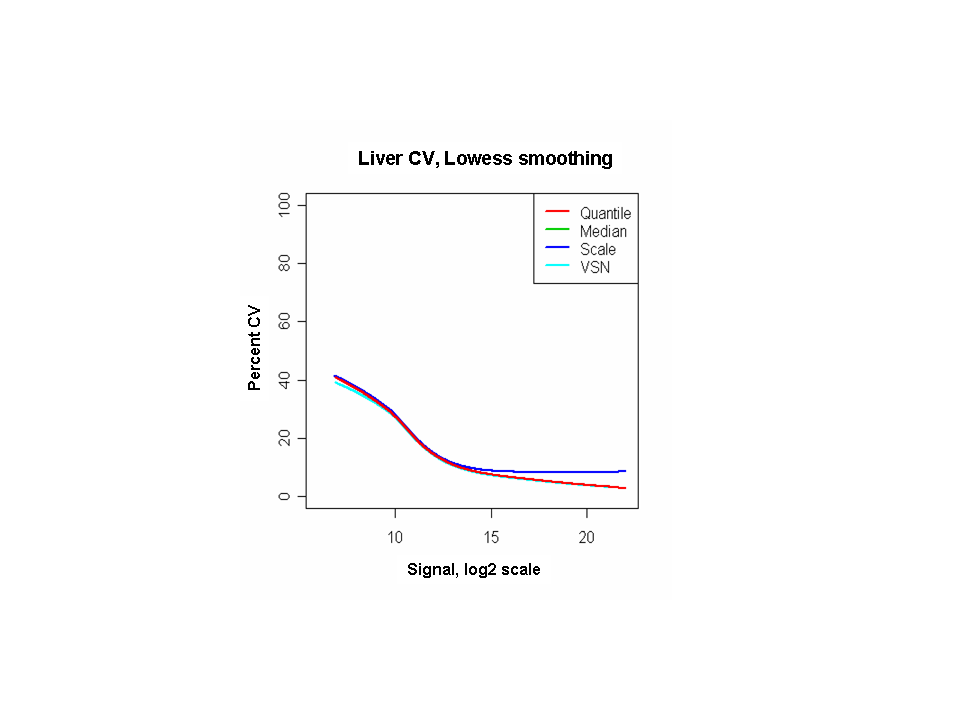

Supplement: Additional File 5 — Comparison of different normalization methods: Coefficients of variation (CV) of technical replicates of Applied Biosystems microarrays. Data on liver sample are shown [8,12]as a representative example. All 33,096 features are represented in this plot. Coefficients of variation (CV) across four technical replicates were calculated using data normalized by different normalization methods, and plotted as a function of average gene expression level. Different normalization methods showed little difference in CV distribution across technical replicates. [file 1471-2164-7-59-S5.png]

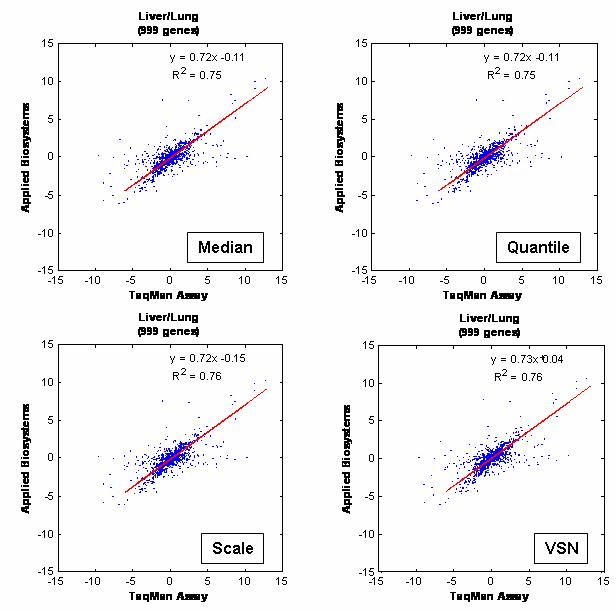

Supplement: Additional File 6 — Comparison of different normalization methods: Correlation of fold change in pair-wise tissues determined by Applied Biosystems microarrays and TaqMan® Gene Expression Assay based real-time PCR. Data on Liver vs. Lung samples are shown as a representative example. y-axis, fold change determined by microarrays which is defined as: log2 (MedianSignal_tissue1/MedianSignal_tissue2); x-axis, fold change determined by real-time PCR, which is defined as ΔΔCt = (Ct_tissue2-Ct_PPIA)-(Ct_tissue1-Ct_PPIA). Genes were filtered based on real-time PCR detection thresholds (detectable in at least 3 out of 4 technical replicates in each tissue and detectable in both tissues, the number of genes are shown in the parentheses). A robust linear regression fitting and the corresponding R2 value are presented in each plot. Different normalization methods showed little difference in fold change correlation with TaqMan assays. [file 1471-2164-7-59-S6.png]

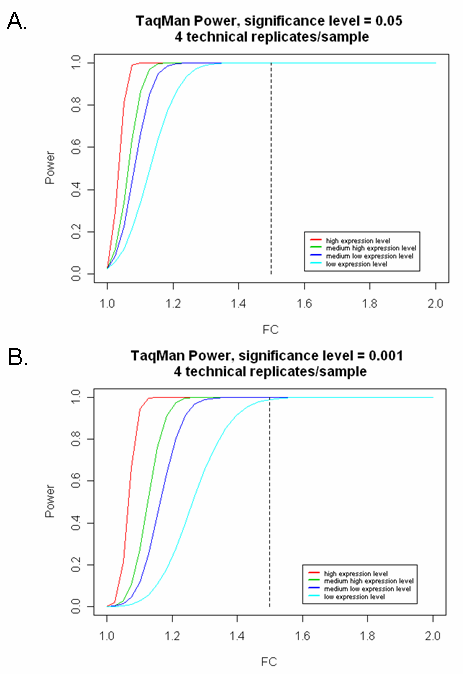

Supplement: Additional File 7 — Power calculation for the TaqMan reference data sets. The power of the TaqMan real-time PCR platform to detect different fold changes using four technical replicates was calculated as described in the Methods for genes with different expression levels. (A). with p-value < 0.05 ; (B). With p-value < 0.001 (equivalent of FDR < 5%). [file 1471-2164-7-59-S7.png]
